# Differences in Arrhythmia Detection Between Harvard Step Test and Maximal Exercise Testing in a Paediatric Sports Population

**DOI:** 10.3390/jcdd12010022

**Published:** 2025-01-11

**Authors:** Massimiliano Bianco, Fabrizio Sollazzo, Riccardo Pella, Saverio Vicentini, Samuele Ciaffoni, Gloria Modica, Riccardo Monti, Michela Cammarano, Paolo Zeppilli, Vincenzo Palmieri

**Affiliations:** 1 Unità Operativa Complessa di Medicina dello Sport e Rieducazione Funzionale, Fondazione Policlinico Universitario Agostino Gemelli IRCCS, Università Cattolica del Sacro Cuore, 00168 Rome, Italy; massimiliano.bianco@policlinicogemelli.it (M.B.); riccardo.pella94@gmail.com (R.P.); saverio.vicentini@gmail.com (S.V.); samuele.ciaffoni01@icatt.it (S.C.); riccardo.monti1@unicatt.it (R.M.); michela.cammarano01@gmail.com (M.C.); paolo.zeppilli@unicatt.it (P.Z.); vincenzo.palmieri@unicatt.it (V.P.); 2 Dipartimento di Neuroscienze, Università Cattolica del Sacro Cuore, 00168 Rome, Italy; gloria.modica@icatt.it

**Keywords:** sport, arrhythmias, pre-participation screening, young athletes, stress test, test modes

## Abstract

BACKGROUND: Sport practice may elevate the risk of cardiovascular events, including sudden cardiac death, in athletes with undiagnosed heart conditions. In Italy, pre-participation screening includes a resting ECG and either the Harvard Step Test (HST) or maximal exercise testing (MET), but the relative efficacy of the latter two tests for detecting arrhythmias and heart conditions remains unclear. METHODS: This study examined 511 paediatric athletes (8–18 years, 76.3% male) without known cardiovascular, renal, or endocrine diseases. All athletes underwent both HST and MET within 30 days. Absolute data and data relative to theoretical peak heart rates, arrhythmias (supraventricular and ventricular) and cardiovascular diagnoses were collected. RESULTS: HST resulted in a lower peak heart rate than MET (181.1 ± 9.8 vs. 187.5 ± 8.1 bpm, *p* < 0.001), but led to the detection of more supraventricular (18.6% vs. 13.1%, *p* < 0.001) and ventricular (30.5% vs. 22.7%, *p* < 0.001) arrhythmias, clustering during recovery (*p* = 0.014). This pattern was significant in males but not females. Among athletes diagnosed with cardiovascular diseases (22.3%), HST identified more ventricular arrhythmias (26.3% vs. 18.4%, *p* = 0.05), recovery-phase arrhythmias (20.2% vs. 14.0%, *p* = 0.035), and polymorphic arrhythmias (6.1% vs. 1.8%, *p* = 0.025). CONCLUSIONS: HST detects arrhythmias more effectively than MET in young male athletes, especially during recovery. More ventricular arrhythmias were highlighted even in athletes with cardiovascular conditions.

## 1. Introduction

The practice of very high-intensity sporting activity, typical of competitive sports, may expose athletes suffering from an unknown cardiovascular disease to an increased risk of serious cardiovascular events, which could sometimes culminate in sudden cardiac death (SCD) [1,2]. The incidence of major cardiac events tends to increase with age [3], and a significant difference in aetiologies has been found between younger and older athletes [4], with congenital heart disease and channelopathies affecting the paediatric sports population the most [5,6].

In Italy, there has long been a mandatory screening protocol, regulated by law, which has proved over the years to have a positive impact on the occurrence of these adverse events [7], although the scientific debate regarding the most cost-effective way of implementing it is still lively and uncertain [8,9]. While the relevance of the resting electrocardiogram (ECG) as an effective screening tool has become increasingly accepted in recent years [10], there is not full agreement on the effectiveness of exercise testing as a diagnostic tool in young athletes. However, to date, there is evidence of the effectiveness of the exercise test in revealing the presence of undetected arrhythmias at rest [11], and this is of utmost relevance given the predictive role determined by the presence of exercise-induced ventricular arrhythmias in cardiovascular mortality [12], particularly in young athletes, in whom SCD arises as a consequence of major ventricular arrhythmias generated, also as a result of the adrenergic stimulus, on a diseased cardiac substrate [13,14].

The exercise assessment in pre-participation screening implemented as per the legal protocol in Italy is based on the execution of a Harvard Step Test (HST), which is characterised by the repetition of ups and downs on a step proportional to the individual’s height at a constant pace for the duration of 3 min; this test is still carried out in this form in the youth population (under 40 years of age), whereas master athletes (over 40 years of age) rely on a maximal ergometric test (MET), which is particularly focussed on detecting signs of inducible myocardial ischaemia [15]. As the most relevant issue in young athletes is to detect exercise-induced arrhythmias that may be triggered by underlying cardiovascular disease, the most effective exercise testing to unveil arrhythmic events should be employed in the paediatric sports population; unfortunately, to date, there are no major data in the literature pointing out the impact of exercise testing modalities on the occurrence of arrhythmic events, so there is no clear evidence to indicate a preference for one or the other test modality (HST vs. MET) for detecting arrhythmias during pre-participation screening. Only one work by Quinto et al. compared these two methods in arrhythmia detection, showing a lower occurrence of ventricular arrhythmias in tests performed with HST than with MET in a young population (between 8 and 35 years of age) of competitive athletes. However, in this work it is emphasised that the electrocardiographic criteria for test maximality were achieved in far fewer individuals in HST than in MET, and on top of that, a comparison was carried out in independent, unpaired samples. Moreover, this article does not clarify the role of the arrhythmic finding in the actual diagnosis of new cardiovascular disease, nor whether there was a difference in the abilities of the tests to point out an underlying heart disease [16]. For all these reasons, we tried to provide useful data to overcome these limitations and to identify the most effective test for uncovering not only arrhythmias but also new diagnoses of cardiovascular disease in a paediatric sport population.

## 2. Materials and Methods

### 2.1. Study Population

This is a retrospective observational study involving athletes who visited our Sports Medicine Unit for examinations aimed at competitive or non-competitive sports eligibility (pre-participation examination, PPE), athletes who came to our practice for a second-level evaluation due to clinical reasons (e.g., electrocardiographic abnormalities, arrhythmias, symptoms) between January 2010 and September 2024 and athletes who participated in prior clinical research protocols.

Paediatric non-competitive and competitive athletes (between 8 and 18 years of age) of both sexes were involved in the study.

All these people were required to perform both HST and TEM on two different days (in order to guarantee full physical efficiency) within one month to reduce confounding factors (e.g., different levels of training, behavioural changes). For the same reason, all participants were instructed not to take any different medication between the tests. Participants were also instructed to report the occurrence of fever, sore throat, gastrointestinal complaints or any symptoms compatible with a possible infection of viral origin, so as to rule out the possibility of intercurrent infections as a trigger for new-onset arrhythmias.

According to the PPE protocol, the following data were collected for every participant:

- Familial history (with a particular focus on cardiovascular diseases);
- Personal history (including cardiovascular risk factors, known cardiovascular diseases and cardiovascular symptoms, particularly if exercise-related, smoking habits, drug consumption, use of energising substances such as caffeine, taurine and alike, medications, allergies, type and intensity of sports practiced, categorised according to the cardiovascular involvement [17]);
- Physical examination (height, weight, arterial blood pressure, cardiac and chest auscultation);
- 12-lead resting electrocardiography (ECG), assessed according to the most recent international guidelines [18]; rhythm abnormalities (brady and/or tachyarrhythmias) and atrioventricular (AV) and intraventricular (IV) conduction abnormalities; pathological Q waves; axis or QRS voltage abnormalities; ST segment or T wave abnormalities;
- Spirometry, with respiratory function indexes in absolute values and as a percentage of the expected values for age and body size (data not reported because they lack relevance);
- HST with continuous ECG monitoring.

Moreover, a MET was performed with cycle-ergometer or treadmill (Cosmed, Albano Laziale, Rome, Italy) based on the aforementioned rules.

The instrumental evaluation sometimes brought to light the presence of misrecognised cardiovascular pathologies, which were noted and classified as follows:

- Bicuspid aortic valve, further distinguished as “near-normal bicuspid aortic valve” and clinically relevant types according to proper definition [19];
- Cardiomyopathies (hypertrophic cardiomyopathy, dilated cardiomyopathy);
- Channelopathies;
- Coronary artery anomalies;
- Major arrhythmias (defined as arrhythmias requiring appropriate diagnostic and therapeutic management, such as atrial fibrillation and supraventricular and ventricular tachycardias requiring invasive assessment with electrophysiological study and ablation);
- Minor congenital heart disease (which included abnormal persistent left superior vena cava, partial venous return, patent foramen ovale, atrial and ventricular septal defects, subvalvular aortic stenosis without haemodynamic significance);
- Mitral valve prolapse, distinguished between minor and moderate/severe/arrhythmic forms;
- Nonischaemic left ventricular scar;
- Ventricular preexcitation.

### 2.2. Inclusion and Exclusion Criteria

Children or adolescents (aged between 8 and 18 years old) of both sexes engaged in recreational, competitive or professional sporting activities were included in the study. They had undergone a sports medicine or sports cardiology assessment including, as a minimum, HST and MET no more than 30 days apart, but on two different days.

The presence of known cardiac pathology, hypertension, endocrine diseases, diabetes mellitus, chronic nephropathy or other known electrolyte disturbances led to exclusion from the study. In addition, the inability to perform one or both types of exercise tests or to achieve at least one of the criteria for maximal testing (see the next paragraph) resulted in exclusion from the study population.

### 2.3. Exercise Testing Assessment

Both the HST and TEM exercise test were performed with the aim of achieving a maximal test; achieving a Rate of Perceived Exertion (RPE) score ≥ 9 on the Borg Category Ratio 10 (CR-10) scale (ranging from 0 to 10) or a maximum heart rate >85% of the expected maximum heart rate for age based on the Cooper formula (220–age) were defined as maximal criteria [20,21].

The HST was performed as an ascent and descent on a platform adapted to the height of each athlete for at least three minutes, or for a longer time until the test maximum was achieved. Where test maximality could not be achieved, the athlete was excluded from the study. ECG monitoring was performed to collect a baseline, continuously for the entire duration of the exercise (at least 3 min in duration as stipulated, and extended until the maximal criteria were met) and for 5 min during the recovery phase, in the supine position.

TEM was performed on a cycle-ergometer or, for children smaller in age and height (who are not able to reach the pedals), on a treadmill, with an incremental ramp or graded protocol customised with the aim of achieving a test duration of between 8 and 12 min, followed as before by a recovery phase duration of 5 min.

Once peak exercise was reached according to previously defined maximal criteria, an abrupt interruption of the exercise phase was carried out in all the different test modes.

The collected data include the peak heart rate, in absolute values and as a percentage of the expected peak heart rate (220–age), test duration, presence or absence of arrhythmias, supraventricular or ventricular origin of arrhythmias, onset at rest, during exercise or after exercise, numerosity, focality, complexity of supraventricular arrhythmias, numerosity, morphology (single morphology or polymorphic and morphology distinguished in “common” and “uncommon” according to Corrado et al. [22]) and complexity of ventricular arrhythmias.

### 2.4. Statistical Analysis

Categorical data were represented as absolute frequencies and percentages (%). The normal distribution of all continuous variables was examined using the Shapiro–Wilk test, and data are presented as the mean ± SD or median (IQR) accordingly. Comparison of means of continuous variables was done using Student’s *t*-test or the Mann–Whitney U test as appropriate, while the McNemar test was used for categorical variables. A two-sided *p*-value ≤ 0.05 was considered to be statistically significant. Jamovi software vers. 2.3.28 was used for statistical analysis.

## 3. Results

The study cohort included 511 mostly adolescent (mean age 14.8 ± 2.4 years) and apparently healthy athletes (390 males, 76.3%). The baseline characteristics, including level of sport practice (competitive or not competitive) and the classification of different sports according to cardiovascular involvement [17] are presented in Table 1.

The exercise testing duration was 191.3 ± 20.9 s for HST and 661.9 ± 169.6 s for MET. The two test modalities (HST and MET) showed a statistically different peak heart rate (PHR) at HST (181.1 ± 9.8 bpm) with respect to PHR at MET (187.5 ± 8.1 bpm) and a different percentage of the expected PHR at HST (88.2 ± 4.7%), percentage of the theoretical PHR at TEM (91.4 ± 3.9%) (*p* < 0.001 for both). A total of 95 athletes (18.6%) showed supraventricular arrhythmias during HST, while 67 (13.1%) showed the same arrhythmias during MET. As for ventricular arrhythmias, these were found in 156 cases (30.5%) during HST and in 116 cases (22.7%) during MET. Upon comparing the occurrence of arrhythmias between the two test methods, a higher incidence of both supraventricular and ventricular arrhythmias was shown in HST than in TEM (*p* < 0.001 for both). Arrhythmias, both ventricular and supraventricular, were subdivided according to the test phase of occurrence as resting, exercise and recovery arrhythmias. Upon comparing the test modalities according to this subdivision, a higher incidence of both supraventricular arrhythmias and ventricular arrhythmias in the HST than in TEM was noted during the recovery phase (*p* = 0.014), whereas this difference was not evident at rest or during exertion.

We also carried out our comparative analysis for each sex, showing the persistence of a statistically significant difference in the prevalence of arrhythmias between the two test modalities in the male sex, whereas this difference was found to disappear when comparing the two in female athletes (Table 2).

**Figure 1:**
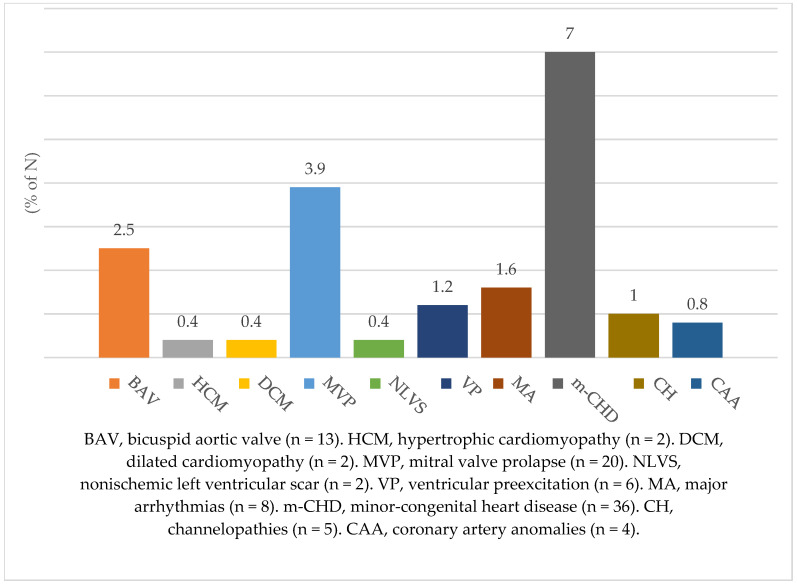
Total number of cardiovascular diseases found in the whole cohort of participants, subdivided on the basis of the resulting pathology (N.B. minor forms of heart disease have been grouped into a single class).

As described in the previous section, all evaluations were part of the diagnostic work-up of the athletes in the cohort, aimed at identifying the potential presence of clinical signs suggestive of cardiovascular disease; this led to the detection of 116 cardiovascular diseases in 114 people (22.3%). Most of these were abnormalities of minimal clinical relevance (e.g., ‘near-normal’ bicuspid aortic valve, non-arrhythmogenic mitral valve prolapse, patent foramen ovale), but clinically relevant cardiovascular diseases such as hypertrophic and dilated cardiomyopathy, nonischaemic left ventricular scar, channelopathies, anomalous origin of coronary arteries, major arrhythmias and ventricular preexcitation were also identified in 29 people (5.6%) (Figure 1).

Among the group of people with cardiovascular diseases, a slight difference in the occurrence of ventricular arrhythmias was found (30, 26.3% in HST; 21, 18.4% in MET, *p* = 0.05), with a significant difference between the two tests for ventricular arrhythmias emerging in recovery (23, 20.2% in HST, 16; 14.0% in MET, *p* = 0.035) and for polymorphic forms (7, 6.1% in HST; 2, 1.8% in MET, *p* = 0.025). No further differences were shown, not even in the subgroup of patients with clinically relevant types of cardiovascular disease (Table 3).

Finally, upon comparing the occurrence of arrhythmias in athletes with cardiovascular diseases to athletes without cardiovascular disease, any significant difference was identified for both supraventricular (22.8% vs. 17.4%, *p* = 0.189) and ventricular arrhythmias (25.4% vs. 32.0%, *p* = 0.181).

## 4. Discussion

This work arose from the need to evaluate the effectiveness of each of the two commonly used exercise test modalities in the PPE (HST and MET) for detecting the presence of arrhythmias. In cardiology, the HST is little used because it does not allow incremental and personalised protocols to be carried out on each patient, and also does not allow the external workload to be estimated correctly. PPE, on the contrary, is considered useful as it allows the athlete to be put under stress quickly but intensively. A problem arises when one has doubts about the efficacy of this test modality, as we pointed out with reference to the work of Quinto et al., who emphasised that HST was less effective at revealing arrhythmias in athletes than the traditional MET [16]. The proposed work aims to address the limitations of the previously mentioned study, at least in part. The primary differences between the two studies that could account for the significant discrepancy in results are that in the current study, a single cohort of individuals underwent both testing modalities, thereby removing the issue of group heterogeneity, which the authors of another study correctly pointed out. Additionally, the peak heart rate (both absolute and as a percentage of the theoretical maximum) achieved in our work during HST was significantly higher, according to the goal mentioned in Materials and Methods Section of achieving at least one criterion of maximality in all tests, even if it means going past the test’s standard 3 min limit and forcing the athletes to go more quickly than the 30 steps per minute target.

Emphasising arrhythmic occurrences is not a goal in itself; rather, it should be used as an early identifier of the existence of an undetected cardiac condition. It is commonly recognised that the sympathetic nervous system being activated (as it is during physical exercise) is a contributing factor to the development of arrhythmias, even though the exact cause is not fully understood. It is, however, recognised that sympathetic activation directly acts on cardiac alpha and beta receptors, increasing heart rate, atrioventricular conduction, and systolic contractility. It also increases the intracellular concentration of calcium ions, which, in a healthy heart, reduces the dispersion of repolarisation, and in a diseased heart can paradoxically lead to an increased dispersion of repolarisation, which often ameliorates major arrhythmic events [23]. In hearts affected by heart disease, an increase in sympathetic innervation and levels of adrenaline and noradrenaline is also common [24], as indirectly demonstrated by the correlation between reduced heart rate variability in chronic cardiovascular diseases and death from major arrhythmic events [25]. For all these reasons, the association between exercise-induced adrenergic activation and major arrhythmias in the presence of a pathological cardiac substrate seems to be the reasonable explanation for all of those events of exercise-induced sudden cardiac death, and justifies the search for arrhythmias as epiphenomena of a potential cardiopathy [26]. Specifically, a growing number of studies in the literature emphasise that high-intensity and continuous protocols may be more useful for detecting arrhythmias than gradual and progressive stress tests, which have not been developed for this specific purpose [27,28]. In light of this, the higher frequency of arrhythmias observed during HST compared to during MET represents a matter of absolute interest and once again reaffirms the strategic importance of this investigation within the PPE, as recently reiterated in the Italian guidelines for eligibility in competitive sports [29].

It is interesting to note that the arrhythmic findings are mainly concentrated in the male sex; this may be due to the lower number of women in our population, but also to the evidence, not yet well explained, of a different development of cardiovascular adaptations to exercise and a lower incidence of cardiovascular disease and sudden cardiac death in female athletes [30]. Certainly, this is an aspect that needs further exploration in the future.

Another important aspect is the prominent role played by the post-exercise recovery period in the induction of both supraventricular and ventricular arrhythmias during HST; this revives interest in the main factors that act as triggers of arrhythmias, with particular reference to the role played by the acute imbalance between the orthosympathetic and parasympathetic nervous systems induced by the abrupt interruption of exercise, as occurred in our patients. In this regard, it is known that a reduced parasympathetic response and/or poor attenuation of sympathetic tone after exercise, manifested as a reduction in heart rate variability, are negative prognostic factors (all-cause mortality) even in relatively young individuals without apparent known heart disease [31]. This aspect, combined with the previously expressed concept that cardiac remodelling in cardiopathies is not only electrical and structural, but also neural, signals the importance of changes in the autonomic nervous system response, both during and after exercise, in the development of arrhythmias, including potentially life-threatening ones [32].

The last point to be mentioned is the higher incidence of ventricular arrhythmias during HST compared to MET in patients identified as having cardiovascular disease; this aspect, although interesting and useful, could be misleading in relation to two other hints that emerge from this work: First, the rate of both supraventricular and ventricular arrhythmias does not appear to be higher in athletes with recognised cardiovascular disease than in those without. According to this, the role of arrhythmic induction during stress tests might be questioned, as the test does not seem to be able to discriminate between athletes with heart disease and healthy individuals; this aspect, interesting as it is, deserves evaluation through a study with a focussed design, eliminating all potential biases related to the specific inclusion criteria of this cohort. Furthermore, once the distinction is made between the cardiopathies we have defined as “minor” and those with greater clinical significance, the difference in the frequency of arrhythmias between the two test modalities disappears; these data must be clearly filtered in view of the small number of subjects with “major cardiopathies” (only 29) and therefore represent an interesting finding, but one that requires a larger sample for a subsequent and more targeted evaluation.

## 5. Conclusions

A higher incidence of arrhythmias has been observed during HST compared to that of MET in a population of young athletes who have undergone both test modalities. These arrhythmias seem to occur mainly during the recovery phase of the exercise. This difference was observed in male participants, but not in females. Moreover, this difference was confirmed, but only for ventricular arrhythmias within the subgroup of individuals with identified cardiovascular abnormalities.

## Figures and Tables

**Table 1:** Baseline characteristics of the population.

Parameters	*N*	Mean	SD
Age (year)	511	14.8	2.4
Height (cm)	511	168.9	12.8
Weight (kg)	511	60.4	14.3
BMI	511	20.9	3.5
Sex:	*N*	% (*N*)	
Male	390	76.3	
Female	121	23.7	
Level of sport participation			
Competitive	446	87.3	
Non-competitive	65	12.7	
Sport according to cardiovascular involvement [17]			
Skill	25	4.9	
Power	31	6.1	
Endurance	75	14.7	
Mixed	384	75.1	

BMI, body mass index. SD, standard deviation.

**Table 2:** Comparison of arrhythmia incidence detected using Harvard Step Test (HST) with that detected using Maximal Exercise Testing (MET) in a cohort of 511 young athletes.

Parameters (*N* = 511)	HST (%)	MET (%)	*p*-Value
PHR > 85% of expected	423 (82.8)	495 (96.9)	<0.001 *
SVPB	95 (18.6)	67 (13.1)	<0.001 *
SVPB-Focality	34 (6.7)	23 (4.5)	0.033 *
SVPB-Rest	8 (1.6)	8 (1.6)	1.000
SVPB-Stress	54 (10.6)	43 (8.4)	0.159
SVPB-Recovery	67 (13.1)	51 (10.0)	0.014 *
SVPB-Complexity	23 (4.5)	14 (2.7)	0.074
VPB	157 (30.7)	116 (22.7)	<0.001 *
VPB-Uncommon	62 (12.1)	49 (9.6)	0.08
VPB-Rest	30 (5.9)	35 (6.8)	0.225
VPB-Stress	95 (18.6)	88 (17.2)	0.473
VPB-Recovery	109 (21.3)	91 (17.8)	0.014 *
VPB-Morphology	24 (4.7)	20 (3.9)	0.465
VPB-Complexity	23 (4.5)	16 (3.1)	0.162
MALE ATHLETES (*N* = 390)			
PHR > 85% of expected	311 (79.7)	377 (96.6)	<0.001 *
SVPB	77 (19.7)	54 (11.5)	0.002 *
SVPB-Focality	26 (15.9)	19 (4.9)	0.071
SVPB-Rest	8 (2.1)	8 (2.1)	1.000
SVPB-Stress	48 (12.3)	36 (9.3)	0.102
SVPB-Recovery	53 (13.6)	41 (10.5)	0.034 *
SVPB-Complexity	17 (4.4)	11 (2.8)	0.134
VPB	121 (31.0)	86 (22.1)	<0.001 *
VPB-Uncommon	51 (13.1)	40 (10.3)	0.109
VPB-Rest	21 (5.4)	23 (5.9)	0.527
VPB-Stress	76 (19.5)	68 (17.4)	0.365
VPB-Recovery	84 (21.5)	66 (16.9)	0.007 *
VPB-Morphology	16 (4.1)	15 (3.8)	0.835
VPB-Complexity	19 (4.9)	11 (2.8)	0.074
FEMALE ATHLETES (*N* = 121)			
PHR > 85% of expected	108 (89.3)	118 (97.5)	0.008 *
SVPB	18 (14.8)	13 (10.7)	0.197
SVPB-Focality	7 (5.8)	4 (3.3)	0.257
SVPB-Rest	0	0	na
SVPB-Stress	6 (5.0)	7 (5.8)	0.705
SVPB-Recovery	14 (11.6)	10 (8.2)	0.206
SVPB-Complexity	5 (4.1)	3 (2.5)	0.317
VPB	35 (28.9)	30 (24.8)	0.197
VPB-Uncommon	11 (9.1)	9 (7.4)	0.480
VPB-Rest	9 (7.4)	12 (9.9)	0.257
VPB-Stress	19 (15.7)	20 (16.5)	0.808
VPB-Recovery	25 (20.7)	25 (20.7)	1.000
VPB-Morphology	8 (6.6)	5 (4.1)	0.257
VPB-Complexity	4 (3.3)	5 (4.1)	0.655

PHR, peak heart rate; SVPB, supraventricular premature beats. VPB, ventricular premature beats. * significant values. na: not available.

**Table 3:** Comparison of arrhythmia incidence detected using Harvard Step Test (HST) with that detected using Maximal Exercise Testing (MET) in athletes with an identified cardiovascular disease.

	HST (%)	MET (%)	*p*-Value
IDENTIFIED CARDIOVASCULAR DISEASE (*N* = 114)			
PHR > 85% of expected	92 (80.7)	108 (94.7)	<0.001 *
SVPB	26 (22.8)	19 (16.7)	0.127
SVPB-Focality	7 (6.1)	6 (5.3)	0.564
SVPB-Rest	3 (2.6)	3 (2.6)	1.000
SVPB-Stress	16 (14.0)	11 (9.6)	0.225
SVPB-Recovery	17 (14.9)	15 (13.2)	0.527
SVPB-Complexity	7 (6.1)	7 (6.1)	1.000
VPB	29 (25.4)	21 (18.6)	0.05 *
VPB-Uncommon	13 (11.4)	10 (8.8)	0.317
VPB-Rest	4 (3.5)	7 (6.1)	0.180
VPB-Stress	18 (15.8)	11 (9.6)	0.071
VPB-Recovery	23 (20.2)	16 (14.0)	0.035 *
VPB-Morphology	7 (6.1)	2 (1.8)	0.025 *
VPB-Complexity	6 (5.3)	5 (4.4)	0.655
IDENTIFIED MAJOR CARDIOVASCULAR DISEASE (*N* = 29)			
PHR > 85% of expected	21 (72.4)	28 (96.6)	0.008 *
SVPB	7 (24.1)	4 (13.8)	0.257
SVPB-Focality	2 (6.9)	2 (6.9)	1.000
SVPB-Rest	0	0	na
SVPB-Stress	3 (10.3)	3 (10.3)	1.000
SVPB-Recovery	6 (20.7)	3 (10.3)	0.180
SVPB-Complexity	1 (3.5)	1 (3.5)	1.000
VPB	5 (17.2)	8 (27.8)	0.180
VPB-Uncommon	4 (13.8)	5 (17.2)	0.564
VPB-Rest	2 (6.9)	5 (17.2)	0.083
VPB-Stress	3 (10.3)	2 (6.9)	0.564
VPB-Recovery	4 (13.8)	5 (17.2)	0.564
VPB-Morphology	3 (10.3)	2 (6.9)	0.317
VPB-Complexity	4 (13.8)	2 (6.9)	0.317

PHR: peak heart rate; SVPB, supraventricular premature beats. VPB, ventricular premature beats. * significant values. na: not available.

## Data Availability

The raw data supporting the conclusions of this article will be made available by the authors on request.
